# Endoscopic Revision for Long‐Term Symptomatic Cage Retropulsion after TLIF: The Clinical Presentation in a Single Center

**DOI:** 10.1111/os.13668

**Published:** 2023-02-14

**Authors:** Guangming Xu, Guangye Zhu, Xiaobing Jiang, Jianchao Cui, Ziyang Liang

**Affiliations:** ^1^ Guangzhou University of Chinese Medicine Guangzhou China; ^2^ Department of Spinal Surgery The First Affiliated Hospital of Guangzhou University of Chinese Medicine Guangzhou China; ^3^ Department of Orthopedics The Second Xiangya Hospital of Central South University Changsha China

**Keywords:** Cage retropulsion, Case report, High‐speed burr, Intervertebral foramen approach

## Abstract

**Background:**

Cage retropulsion after transforaminal lumbar interbody fusion (TLIF) is a common complication that is more frequently detected in the early postoperative period. Revision in the early stages is relatively less difficult in symptomatic cases. However, cage retropulsion is quite rare for patients with intervertebral osseous fusion in the long term after TLIF, and there are no relevant reports related to the revision plan.

**Case presentation:**

Here, we report a case of a patient who underwent L4‐S1 TLIF at another hospital 4 years ago, accompanied by recurrent pain and discomfort of the left lower limb after the operation. Due to recent condition aggravation, it was considered to be caused by compression of the nerve root due to cage retropulsion. Nerve root sealing and endoscopy surgery were performed on the operative segment. It was found that cage retropulsion at the L4/5 level was a suspicious focus according to careful analysis of the clinical manifestations of the patient. Selective block of the nerve root on the level resulted in relief of the patient's original symptoms. After the posterior edge of the cage was exposed under the endoscope through an intervertebral foramen approach, the posterior edge of the cage protruding into the spinal canal was removed by high‐speed burr grinding, working casing reduction and other methods. Postoperative symptoms of pain in the low back and lower limb were relieved completely.

**Conclusions:**

It is feasible to use the power system to remove the retrograde cage under the endoscope through the intervertebral foramen approach for the revision of symptomatic polyether ether ketone (PEEK) cage retropulsion in the long term after TLIF.

## Graphical abstract

The implantation of interbody cage can not only restore the height of interbody in time, but also provide a stable bone grafting bed, supplying enough guarantee, for the short‐term and long‐term stability respectively. An open surgery is required in the case of nerve compression and decrease in stability after the displacement of the cage.

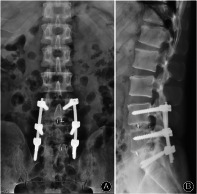

## Background

Posterior lumbar interbody fusion (PLIF) and TLIF are safe and effective methods for the treatment of lumbar degenerative disease. The implantation of interbody cages can not only restore the height of the interbody in time but also provide a stable bone grafting bed, guaranteeing short‐ and long‐term stability.^1^, ^2^, ^3^ However, new potential complications, including displacement of the cage and collapse of the intervertebral space, have drawn attention to the implanted cage. Open surgery is required in the case of nerve compression and stability decrease after displacement of the cage.^4^, ^5^ However, for patients undergoing long‐term revision, the difficulty of surgery and the risk of nerve injury will be increased due to a large number of resultant scars surrounding the spinal nerve.

## Case Presentation

**Fig 1:**
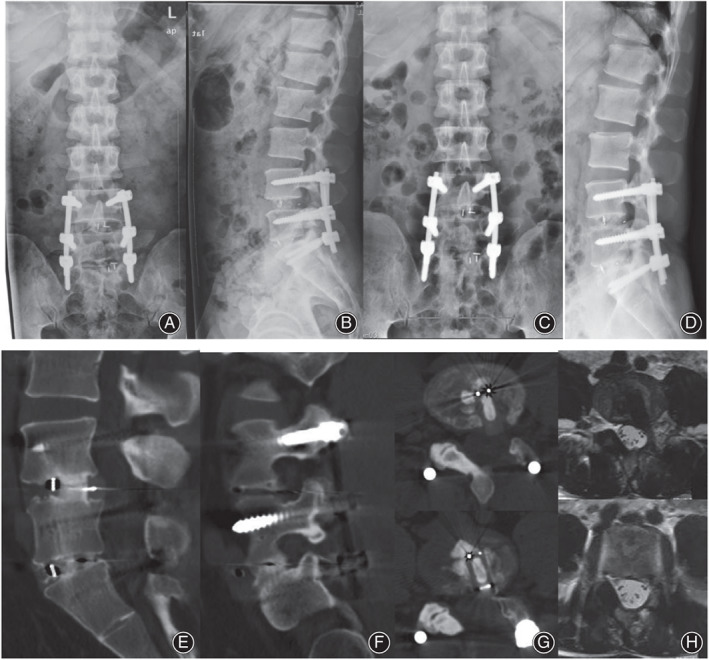
Imaging material of pre‐ and post‐operation. (A, B) Postoperative anterioposterior and lateral radiographs after L4/5 and L5/S1 TLIF in 2014; (C, D) X‐ray of lumbar spine in the patient on admission of our hospital in 2018; (E–G) CT of lumbar spine in the patient on admission of our hospital in 2018; (H) MRI of lumbar spine in the patient on admission of our hospital in 2018

A 48‐year‐old male patient underwent L4/5 and L5/S1 TLIF in another hospital due to “low back pain” in 2014 (Fig. Fig 1A, B). The patient showed low back pain that disappeared postoperatively but was accompanied by radiative pain in the left lower limb that recurred occasionally, with normal muscle strength. After TLIF, the patient had been taking anti‐inflammatory and analgesic drugs. However, 3 months prior, the patient developed recurrent lower back pain after bending and lifting heavy objects, accompanied by numbness, which became worse while walking and was relieved while lying in the supine position. Physical examination of the patient showed 40° (+) in the straight leg raising test of the left limb, positive (+) result of the augmentation test, decreased acupuncture propagated sensation in the left L5 dermatome, and extensor muscle strength grade IV of the left hallux dorsum.

In 2018, an X‐ray of the patient on admission to our hospital showed no obvious loosening of the original internal fixation screws. MRI of the patient on admission to our hospital showed stenosis of the left lateral recess in the 4/5 space of the lumbar spine (Fig. Fig 1H). The posterior part of the interbody cage in the 4/5 space of the lumbar spine protruded into the spinal canal, with stenosis of the left lateral recess of the corresponding level (Fig. Fig 1E–G). The patient experienced aggravated pain when extended and flexed backward, difficulty standing on one leg of the left lower limb and poor elevation outcome of the left straight leg.

The following is the diagnosis and treatment process as well as the technical introduction of the patient.

**Fig 2:**
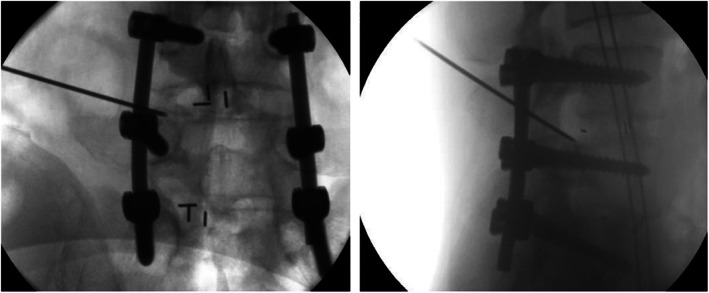
Fluoroscopy image of the puncture needle when performing the nerve root block

Combined with the imaging data, it was considered that the adverse symptoms might be related to the nerve compression caused by the position deviation of the cage. For the identification of the source of symptoms, a L5 nerve root block was conducted in the left L4/5 space (Fig. 2). After injection of 1 ml diluted lidocaine, the original lumbar pain and radiating pain in the left lower limb completely disappeared.

On the first day after the block, radiative pain occurred again in the lower back and left lower limb of the patient. In view of a definite diagnosis, a treatment plan was made for percutaneous transforaminal endoscopic L4/5 lateral recess decompression.

**Fig 3:**
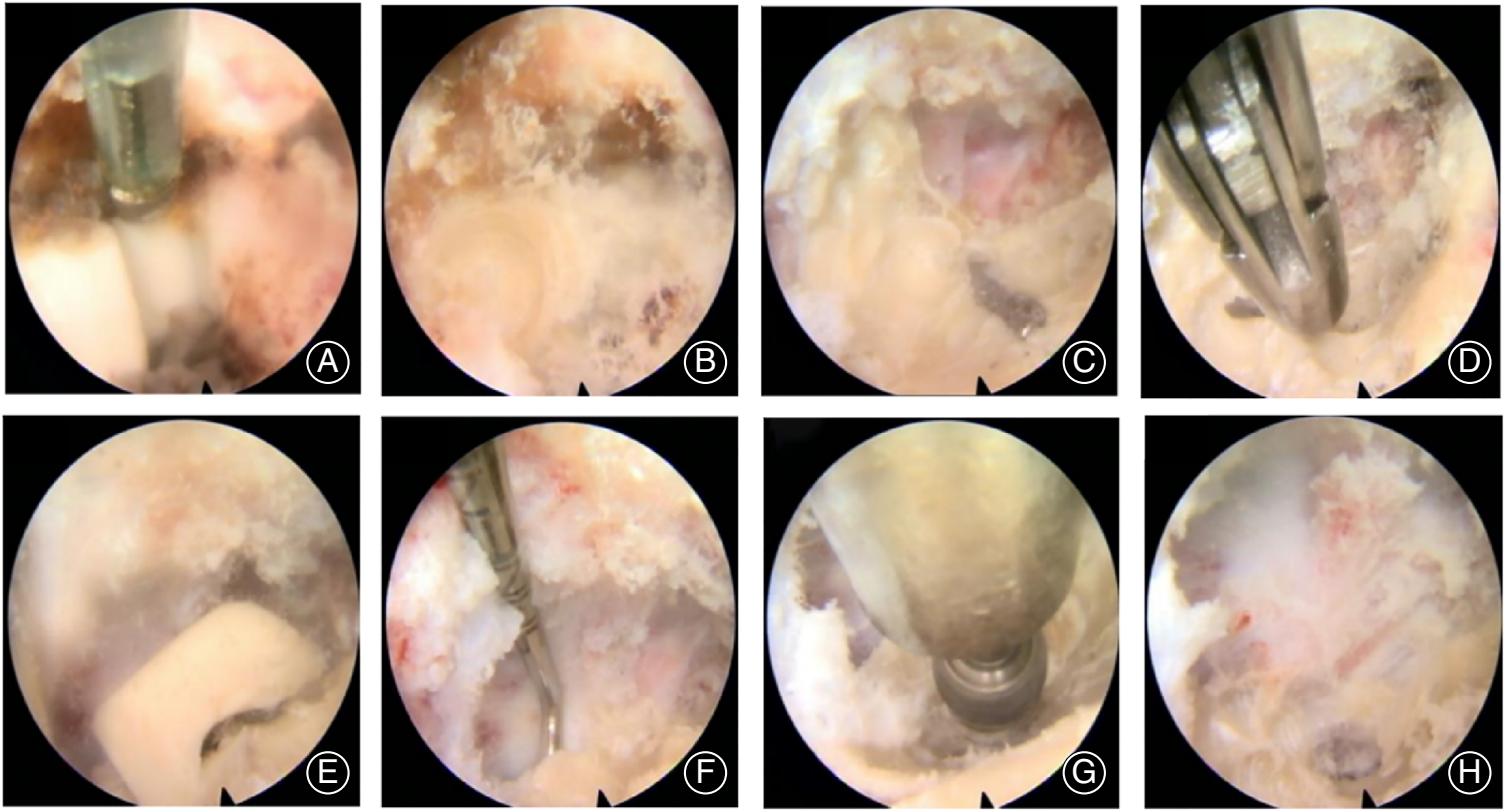
Operation procedures under the endoscope. (A) Posterior edge of the cage was higher than vertebrae. (B) Cage surface with worn. (C) Taken out the soft tissue structure; (D, E) Removed the residual upper corner of the cage. (F) Used probe hook to detect the thickness of the residual cage. (G, H) Grind the residual cage to become thinner

With the lateral recess structure exposed, soft tissues in the spinal canal were cleaned by radiofrequency. The posterior edge of the cage was shown to be higher than the posterior vertebral body (Fig. 3A). Subsequently, the rear part of the cage was ground using a grinding drill under the endoscope, and the cage surface was observed to be worn (Fig. 3B). The grinding range was then expanded and exposed to the inner edge of the cage. The inner posterior edge of the cage was broken, and the exposed soft tissue structure was removed (Fig. 3C). The residual upper corner of the cage after grinding was broken by rotating the sleeve (Fig. 3D, E). A probe hook was then used to detect the thickness of the residual cage on the inner edge (Fig. 3F), and the grinding drill was continued until the residual part became thinner (Fig. 3G, H).

After the operation, the patient achieved complete relief of lower back pain and radiating pain in the lower limb.

**Fig 4:**
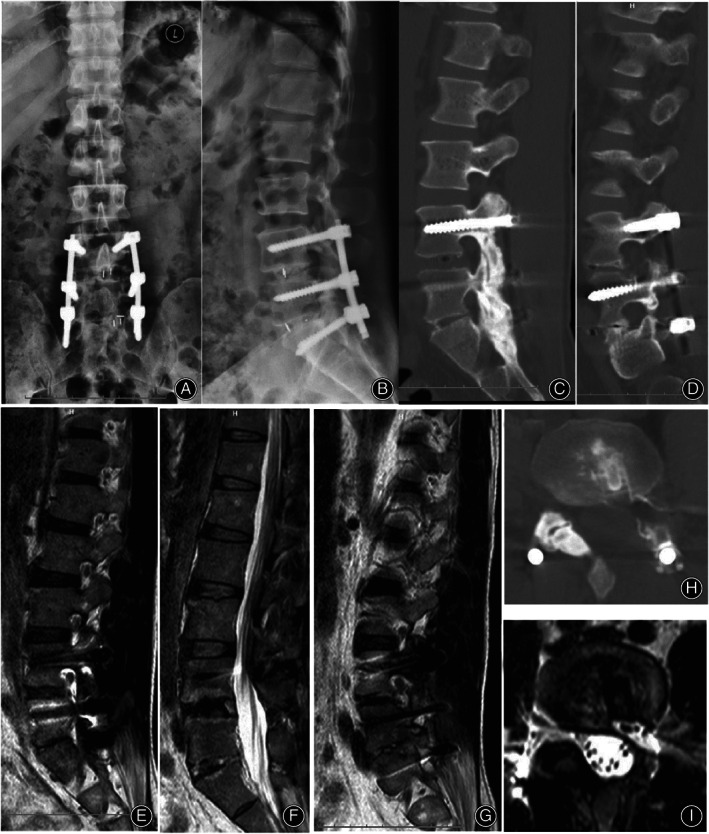
Imaging material of post‐operation this time. (A, B) Postoperative X‐ray review; (C, D, H) Postoperative CT scan review; (E–G, I) Postoperative MRI review

As shown in Fig. 4A, B, postoperative X‐rays showed no loosening or displacement of the original implant, and the L4/5 posterior edge marker disappeared. In Fig. 4C, D, H, postoperative CT showed that the posterior edge of the cage was abraded, and the left superior articular process of L5 was partially removed, forming a bone tunnel. Postoperative MRI review showed the removal of part of the left superior articular process of L5 and enlargement of the space of the left intervertebral foramen and lateral recess of L4/5 (Fig. 4E–G, I). The patient was satisfied with the curative effect, with complete disappearance of low back pain and radiative pain in the left lower limb.

## Discussion

Cage displacement is a common complication in interbody fusion.^6^ As reported in the past, a majority of patients who need opening repair due to the problem of cage retropulsion usually occurring within 3 months after the first operation, with less scar adhesion and no interbody fusion at that time, show a small technical difficulty in the operation.^7^ Theoretically, lumbar spinal fusion surgeries involved the risk of fusion cage retropulsion. The endoscopic surgery can allow minimal blood loss and focused neural decompression to reduce the complication.^8^, ^9^, ^10^ However, it should be noted that for patients with neurological symptoms resulting from poor positioning of the cage and interbody fusion, in most cases there are few reports concerning the technique of its revision in the literature.

In our case report, an X‐ray taken 1 week after surgery in the first hospital indicated a slight retrodisplacement of the cage. Considering no change in the range of radiative pain in the lower limb, but with aggravated degree only, it was preliminarily judged that the responsible focus was still related to cage retropulsion at L4/5.^11^ To identify that the proposed segment was responsible, a root block test at the L4/5 segment was performed first, and the patient immediately reported the disappearance of symptoms after the block. However, the original symptom appeared again the next day, and it could thus be clarified that the discomfort was caused by cage retropulsion.^12^

In consideration of avoiding the scarring effect of the original approach, a scheme of endoscopic removal of the cage was developed *via* the intervertebral foramen approach under local infiltration anesthesia. Following conventional location puncture and endoscopic‐assisted decompression of the lateral recess with the power system under the endoscope, the edge of the protuberant cage was exposed through the endoscope beyond the posterior wall of the vertebral body. Subsequently, the cage was removed through an outside‐in approach by using a high‐speed grinding drill under the endoscope until entering the intervertebral space, and the retrograde cage was disconnected at the head‐tail ends and inside‐outside within the intervertebral space. In the next step, part of the marginal cage was compressed and removed. Pain and discomfort in the low back and lower limb were induced repeatedly during the entire process of grinding. However, when the cage protruding into the spinal canal was completely removed, the dural sac and nerve root were observed to float freely with the scar tissue under the change in water pressure. The patient informed the surgeon that numbness and pain in the lower limb had disappeared completely. At present, the patient has received revision for over one year, with good follow‐up results.

After defining the responsible segment in our case, the difficulty of decompression through the posterior median approach definitely increased considering the presence of cage retropulsion combined with the formation of a scar in the spinal canal.^11^ Simultaneously, CT showed that there was solid bone fusion in and around the cage, associated with great trauma when removing the cage again and a high risk of nerve injury. Thus, after knowing the material properties of the cage (PEEK) and the location of the cage retropulsion in the medial intervertebral foramen parallel to the intervertebral space, it was considered that a high‐speed grinding drill could be used to remove the retrograde cage. Meanwhile, through Kambin's triangle of the intervertebral foramen and the formation of the intervertebral foramen, an angle and operation space could be constructed for the endoscopic canal to avoid the dural sac of the nerve root and directly reach the compression focus, which was more conducive to the completion of revision and decompression. Our study also proves that the high‐speed power under the endoscope can effectively remove the implant of PEEK material. In addition, considering complete fusion of the bone structure in the intervertebral space, the edge of the cage protruding from the posterior edge of the vertebral body may have no influence on the stability of the intervertebral body after grinding. Collectively, it is feasible to use the power system to remove the retrograde cage under the endoscope through the intervertebral foramen approach for the revision of symptomatic PEEK cage retropulsion in the long term after TLIF.

### Conclusion

It is feasible to use the power system to remove the retrograde cage under the endoscope through the intervertebral foramen approach for the revision of symptomatic PEEK cage retropulsion in the long term after TLIF.

## Author Contributions

Guangming Xu: Manuscript writing. Guangye Zhu: Data analysis and interpretation. Xiaobing Jiang: Collection data. Ziyang Liang and Jianchao Cui: Conception and design.

## Funding Information

This work was generously supported by Scientific Research Project of Excellent Young Scholars Project of the First Affiliated Hospital of Guangzhou University of Chinese Medicine (Grant No. 2019QN17), Scientific Research Project of Traditional Chinese Medicine Bureau of Guangdong Province (Grant No. 20201097), Natural Science Foundation of Hunan Province (Grant No. 2022JJ40696) and Natural Science Foundation of Changsha City (Grant No. kq2202390).

## Ethics Statement

The institutional review board of the local hospital approved this study (NO. ZYYEC‐ERK [2020] 019).

## Consent for Publication

Written informed consent for publication of this case report and any accompanying images was obtained from the patient.

## Data Availability

The datasets used during the current study are available from the corresponding author on reasonable request.
